# Osseointegration of Tantalum Trabecular Metal in Titanium Dental Implants: Histological and Micro-CT Study

**DOI:** 10.3390/jfb14070355

**Published:** 2023-07-06

**Authors:** Modhi Al Deeb, Abdullah AlFarraj Aldosari, Sukumaran Anil

**Affiliations:** 1 Department of Prosthetic Dental Science, College of Dentistry, King Saud University, P.O. Box 60169, Riyadh 11545, Saudi Arabia; 2 Department of Dentistry, Oral Health Institute, Hamad Medical Corporation, Doha P.O. Box 3050, Qatar

**Keywords:** dental implants, osseointegration, titanium, X-ray microtomography, histology, bone regeneration, bone remodeling, titanium–tantalum implant, trabecular metal, dentistry, implantology

## Abstract

This study aimed to investigate the impact of the Tantalum Trabecular Metal dental implant design on implant stability and the process of osseointegration following its placement in the rabbit femoral condyle. The subjects for the experiment consisted of 10 New Zealand white rabbits. Twenty implants, comprising 10 Trabecular Metal (TM) and 10 Traditional Screw Vent (TSV) implants, were placed into the femoral condyles of these rabbits. The implant type was alternated based on a random sequence. Following a healing period of 8 weeks, the implants were retrieved for further analysis using micro-computed tomography (micro-CT), histological studies, and histomorphometry evaluations. The Bone-to-Implant Contact (BIC) ratio and the Bone Volume (BV) percentage in the region of interest were subsequently assessed. The BIC and BV values between TM and TSV implants were compared using the Student *t*-test. The TM implants exhibited significantly greater BIC and BV scores. In particular, the BIC percentage was recorded as 57.9 ± 6.5 for the TM implants, as opposed to 47.6 ± 8 for the TSV implants. Correspondingly, the BV percentage was 57 ± 7.3 for the TM implants and 46.4 ± 7.4 for the TSV implants. The bone volume percentage measured using micro-CT evaluation was 89.1 ± 8.7 for the TM implants and 79.1 ± 8.6 for the TSV implants. Given the observed results, it is plausible to suggest that the bone growth surrounding the tantalum mesh could have improved the integration of the bone and facilitated its ingrowth into the TM implant.

## 1. Introduction

Dental implants have dramatically transformed oral rehabilitation, presenting an effective solution for partially and completely toothless patients. Although there have been reports of high success rates with implant-supported prostheses, a small portion still fails [1,2,3,4]. Improvements to implant surfaces have been reported to improve bone integration [5,6], and continuous efforts are being made to enhance the success and survival rates of dental implants [7,8].

Porous Tantalum Trabecular Metal (PTTM) is a material that has been used in dental implants due to its ability to increase surface roughness and promote osseointegration, which is the direct growth of bone into the implant [9]. PTTM is 80% porous, which is similar to that of bone microstructures, and it also has similar elasticity [10]. The trabecular part of the implant increases the surface area and promotes osseoincorporation through bone ongrowth and ingrowth [11]. Studies have shown that titanium encourages cell proliferation, while tantalum promotes the osteoblastic differentiation process [12,13]. The porous structure of tantalum, similar to that of spongy bone, is thought to be a factor in promoting bone ingrowth [9,14]. PTTM has also been used in orthopedic implants due to its ability to enhance neovascularization, wound healing, and osteogenesis [11]. Overall, the use of PTTM in dental implants offers potential benefits for promoting bone integration and the long-term success of the implant [13,15].

The Trabecular Metal dental implant combines tantalum and titanium alloy to enhance functionality and osseointegration [14,16]. The midsection of the implant is made from a porous tantalum meshwork, which allows for neovascularization and new bone formation directly into the implant [11,17,18]. The coronal and apical sections of the implant are screw-shaped and made from a titanium alloy [19]. The titanium alloy surfaces undergo micro texturing with hydroxyapatite to enhance osseointegration [19]. The Trabecular Metal dental implant has a similar structure to trabecular bone, allowing for improved biomechanical properties and long-term stability [9,20].

While previous research has offered some insights into the healing patterns, integration, and bone response with the Trabecular Metal dental implant, our understanding of these processes specifically in the PTTM-enhanced areas of the implant remains incomplete [5,21]. The current study aims to address this knowledge gap by comparing the Trabecular Metal dental implant to an experimental implant system from the same manufacturer. We employ advanced techniques, including micro-CT and histomorphometry, for a more detailed and comprehensive analysis of bone volume and bone-to-implant contact. These methods will contribute to a more nuanced understanding of the osseointegration process with the Trabecular Metal dental implant. Therefore, our objective is to explore bone healing around the PTTM-enhanced dental implant in a rabbit femoral condyle and contrast these findings with the experimental implant system.

## 2. Materials and Methods

In accordance with the ARRIVE guidelines, the study employed 10 New Zealand White rabbits, aged between 6–9 months, with weights varying from 3.5 to 5 kg. The rabbits were sourced from the central experimental animal research facility at King Saud University. The sample size was determined based on an a priori power analysis to minimize the number of animals used while ensuring statistically significant findings. The rabbits were individually housed in standard cages with a 12-h light/dark cycle, at a controlled room temperature (22 ± 2 °C) and relative humidity (55 ± 10%). The cages were furnished with soft bedding and environmental enrichment items, such as chew toys and tunnels, to promote natural behavior [22,23].

The rabbits were fed twice a day with commercially available rabbit food pellets, supplemented with fresh vegetables for additional hydration and nutrients. Fresh drinking water was provided ad libitum. Before the initiation of the study, rabbits were given a two-week acclimatization period to adjust to the environment and handling. The rabbits’ health was regularly monitored, with veterinary care available as needed.

The study protocol involving animals was reviewed and approved (NF2322) by the Animal Ethics Committee at the College of Dentistry, King Saud University in Riyadh, Saudi Arabia, which ensured adherence to all national standards for the care and use of laboratory animals. The bilateral rabbit femoral implant model served as a control for the experiment. 

### 2.1. Implants

**Figure 1:**
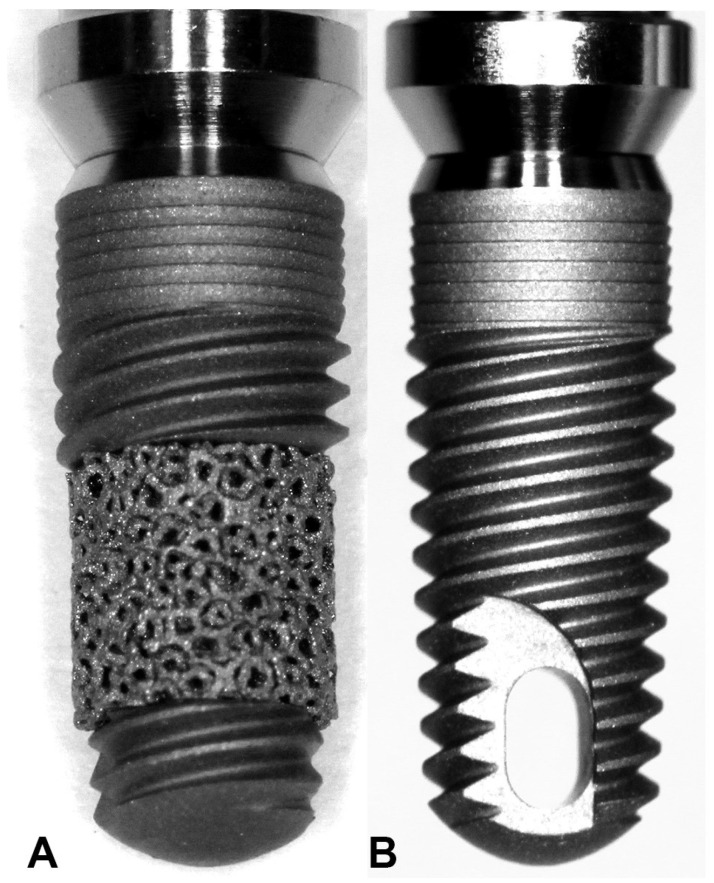
This figure showcases the two implant systems employed in this research—the Trabecular Metal implant (**A**) and the Screw Vent implant (**B**).

Twenty commercial implants of two types were employed for this study: Trabecular Metal ™ implants (Trabecular Metal^®^ Implant, Zimmer Dental Inc., Carlsbad, CA, USA), and Tapered Screw Vent™ implants by Zimmer (Tapered Screw Vent ^®^ Implant, Zimmer Dental Inc., Carlsbad, CA, USA). Both types of implant had dimensions of a 4.1 mm diameter by 10 mm length (Figure 1).

Surgical Procedures: The surgical procedures were carried out under strict sterile conditions coupled with general anesthesia initiated by intramuscular injections of a ketamine mixture (35 mg/kg, Parke-Davis, Morris Plains, NJ, USA) and a xylazine dose of 5 mg/kg. Post-anesthesia, the hind limbs of the rabbits were shaved, cleaned, and draped to isolate the surgical area, followed by local anesthesia administration at the operative sites. Medial parapatellar longitudinal incisions were made using a BP Blade #15 (Swann Morton, Sheffield, UK) to expose the left and right knee joints. 

The patella was laterally displaced to reveal the medial femoral condyle, which required incising through the capsule. Dental drills were used to bore a hole through the articular cartilage into the subchondral bone situated on the weight-bearing surface of the femoral condyle. This drilling procedure followed the manufacturer’s instructions, incorporating external cooling with sterile saline. The implant bed was expanded in stages, starting with a pilot drill and proceeding to 2.3 mm, 2.8 mm, 3.4 mm, and finally to 3.8 mm in accordance with the company’s surgical protocol (W&H Dentalwerk Bürmoos GmbH by Zimmer Dental Inc., Carlsbad, CA, USA).

**Figure 2:**
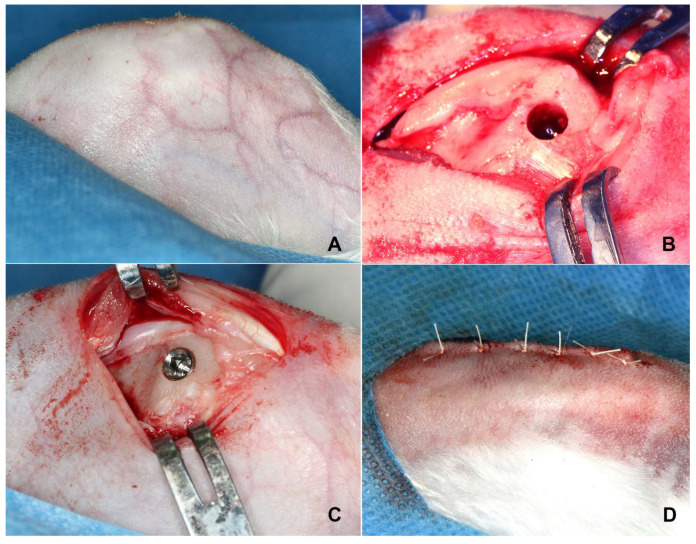
This figure illustrates the surgical procedure (**A**) the medial surface of the femoral condyle with the skin and fascia, (**B**) the drilled hole in the femoral condyle, (**C**) the implant in its proper position, and (**D**) the surgical site after it has been closed and sutured.

Following a randomization protocol, each rabbit received alternating implant types in the right and left femur. The implants were inserted according to a pre-determined randomized sequence (Table 1, Figure 2C,D) [24]. Resorbable sutures (Vicryl, 4-0) were used to close the surgical sites, and the animals were returned to their cages. All efforts were made to minimize any discomfort or distress during the procedure, including the administration of analgesics for post-operative pain relief. The implant site was regularly monitored for signs of infection or complications. Post-operative pain was managed with intramuscular doses of non-steroidal anti-inflammatory drugs (NSAIDs), and to minimize the risk of post-operative infection, Enrofloxacin (5–10 mg/kg, Baytril, Bayvet Division, Chemagro Ltd., Etobicoke, ON, Canada) was administered. Post-implantation, the rabbits were monitored daily for any behavioral changes, discomfort, or health complications. Following an eight-week post-implantation period, the animals were euthanized under deep anesthesia and the femoral condyles were harvested for micro-CT and histological examination.

### 2.2. Micro-Computer Tomography

Following fixation in formaldehyde and dehydration in 70% ethanol, the bone samples underwent three-dimensional micro-computed tomography (micro-CT) to determine the bone mineral density and volume around the implant. During scanning, the specimens were wrapped in Parafilm M^®^ (Pechiney Plastic Packaging, Chicago, IL, USA) to prevent drying. The samples were then scanned at an energy level of 101 kV and intensity of 96 μA with a resolution of 37.41 μm per pixel, using an aluminum filter (1 mm) (Skyscan-1072 X-ray Microtomograph, TomoNT version 3N.5, Skyscan^®^, Kontich, Belgium). Bone mineral density calibration rods were also scanned as a reference. Cone-Beam reconstruction was performed using Skyscan^®^ software (version 2.15, Bruker MicroCT, Kontich, Belgium). All scan and reconstruction parameters were standardized for the specimens and calibration rods [25].

The data were analyzed using the CT Analyzer (version 1.4, Skyscan^®^). The region of interest (ROI) was defined as an annular area with a diameter of 1.0 mm surrounding the implants, extending 3 mm in length. Bone mineral density (BMD) and bone volume (BV) within this area were calculated and expressed as percentages. BMD was defined as the amount of bone mineral per unit volume of bone tissue (g/cm^3^) and calibrated using calibration rods with known BMD (0.25 g/cm^3^ and 0.75 g/cm^3^), and a Hounsfield Unit calibration for water and air density. The mean (total) value for density, representing an average of trabecular bone and bone marrow, was used to denote the bone mineral density of the trabecular bone around the implants, as recommended by Skyscan^®^. BV (mm^3^) was expressed as a percentage of the total ROI volume [26].

### 2.3. Histomorphometric Evaluation

Post-euthanasia, the femoral condyles were collected for histological processing, and a histomorphometric evaluation (percentage of bone-to-implant contact [BIC percent] evaluation) was performed. The femoral condyles, along with the implants, were fixed in 10% formaldehyde. The specimens were then reduced in size, dehydrated in escalating ethanol concentrations (70–100%), and finally embedded (non-decalcified) in modified methylmethacrylate (MMA) for five days. Longitudinal sections (10 μm) were then cut in the mesiodistal direction relative to the implant axis using an inner circular saw microtome (Leica RM 1600, Nussloch, Germany). These sections were stained with methylene blue and basic fuchsin for examination under a light microscope and subsequent histomorphometric analysis.

The automated Zeiss Z1 Axio Imager light microscope (Carl Zeiss MicroImaging GmbH, Göttingen, Germany) was employed for the histological examination. Histomorphometry was conducted utilizing digital image analysis software (Leica^®^ Qwin Pro-image analysis, Cambridge, UK). Two quantitative parameters were analyzed: the percentage of bone in contact with the implant (BIC percent) and the bone volume (BV percent).

BIC: Bone contact was analyzed in an area from the first thread of the implant extending up to 6 mm. BIC was defined as the percentage of the implant surface in direct contact with bone without an intervening fibrous tissue layer. Bone contact was then expressed as a percentage of the total bone contact over the 6 mm implant length. All measurements were performed on the implant on three histological sections per implant. The data from these three sections for each implant was averaged.

Percentage of peri-implant bone area (BV): The percentage of bone present within the selected region of interest (ROI), extending 6 mm from the first thread, was considered. BV measurements were based on quantifying the bone tissue in this ROI, which was set as a virtual cylinder. All measurements were made on both sides of the implant for three histological sections per implant.

### 2.4. Statistical Analysis

All statistical analyses were conducted using GraphPad^®^ Instat 3.05 software (GraphPad Software Inc., San Diego, CA, USA). The BIC and BV values between TM and TSV implants were compared using the Student *t*-test. Differences were considered statistically significant at *p*-values of less than 0.05. 

## 3. Results

### 3.1. General Observation

All test subjects maintained good health throughout the eight-week testing period, with no indications of infection or discomfort. The sites of implantation healed devoid of any signs of inflammation or infection. A gross examination of the retrieved samples revealed that each implant was adequately positioned.

### 3.2. Histology

**Figure 3:**
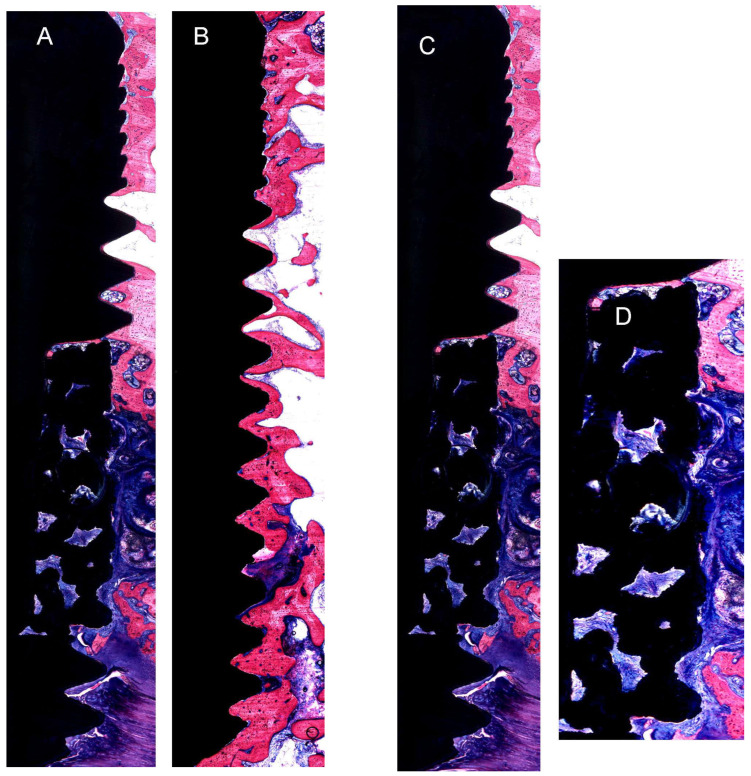
This figure presents representative histological images of the interface between the implant and bone following an 8-week healing. (**A**) Trabecular Metal Implant (TM) and (**B**) Screw Vent Implant (TSV). (**C**,**D**) Trabecular Metal Implant showing the area of tantalum Trabecular area (Objective x10).

Gross Histological Observation: Detailed microscopic examination of the implant and adjacent tissue sections, stained with methylene blue/basic fuchsin, revealed bone apposition, remodeling, and the proliferation of newly formed bone on the implant surface across all samples. A dense concentration of osteocytes, which had adhered to the implant’s surface, was observed in the bone close to the implants. A dense layer of lamellar trabecular bone was evident on the surface of both the TM and TSV implants, as seen in Figure 3.

Histological Observation: The results of histological analyses revealed a higher quantity of bone surrounding the implant, adhering more closely to the trabeculae than to the threaded surfaces of the titanium alloy (Ti) (Figure 3A,B). The newly formed bone, originating from the remnants, proliferated into thick, short trabeculae within the tantalum framework, making contact with the metal. The tantalum mesh area displayed enhanced bone growth and increased infiltration of osteocytes as observed histologically. The formation of vascular canals and partial remodeling of the bone trabeculae were noted. These bone trabeculae were densely populated with osteocyte lacunae, the majority of which were spherical and irregularly distributed, a typical feature of woven bone (Figure 3C,D).

**Figure 4:**
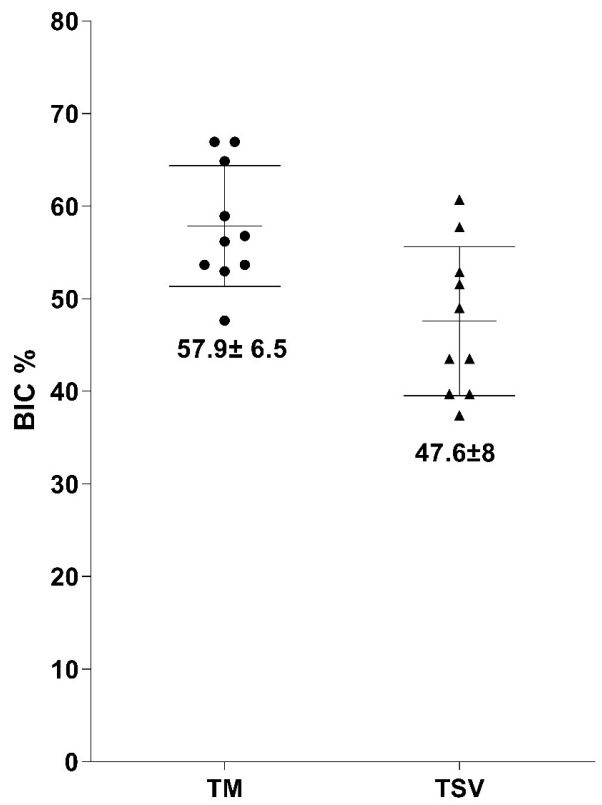
This figure presents a graphical depiction of the Bone-to-Implant Contact (BIC) percentage for both the Trabecular Metal Implant and the Screw Vent Implant (TSV).

**Figure 5:**
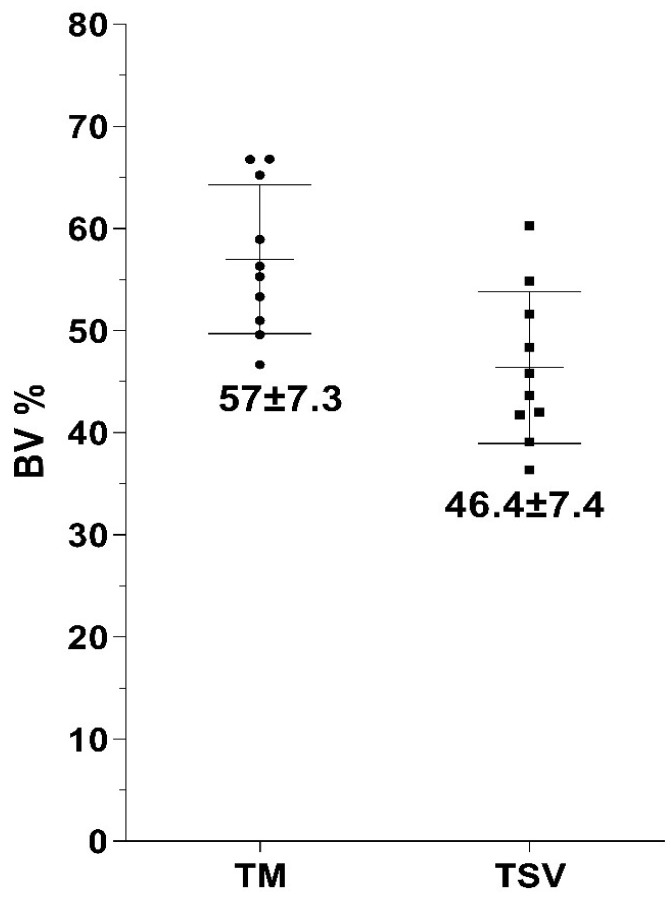
This figure displays a graph highlighting the Bone Volume (BV) percentage in the region of interest surrounding both the Trabecular Metal Implant and the Screw Vent Implant (TSV).

Bone-to-Implant Contact Percentage (BIC%): The BIC percentages for both TM and TSV implants and the average and standard deviation values are depicted in Figure 4. Initial bone contact measurements revealed substantially greater BIC values for TM implants compared to the control TSV implants. The percentage of direct bone-to-implant contact (BIC) along the length of the implant in the region of interest was recorded as 57.9 ± 6.5 for TM implants and 47.6 ± 8 for TSV implants. After a healing period of eight weeks, the TM implant group’s BIC was significantly higher than that of the TSV implant group (control), as depicted in Figure 5.

Bone Volume Percentage (BV%): The BV percentages obtained from the histological sections echoed the BIC scores with an average of 57 ± 7.3% for the TM group and 46.4 ± 7.4% for the TSV group (Figure 5). The percentage of bone growth within the TM implant in the region of interest was superior to that of the TSV implant. The TM implants demonstrated a significantly larger volume of bone ingrowth in the tantalum midsection compared to the control TSV implants. The results from the BIC and BV measurements suggest that the TM implant significantly influenced the amount of bone contact with the implant surfaces and the volume of bone ingrowth in the central trabecular area.

**Figure 6:**
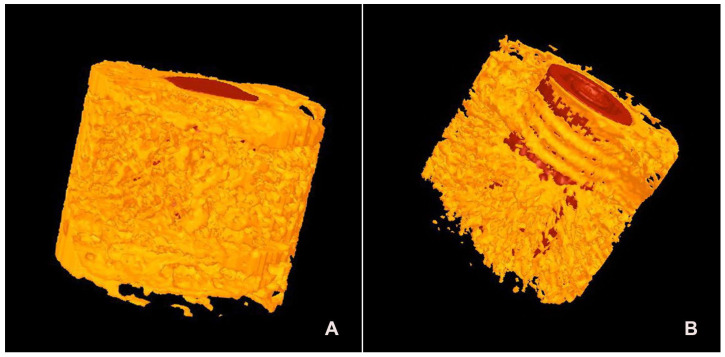
This figure displays micro-CT images of three-dimensional reconstructed sections for both the Trabecular Metal Implant (TM) (**A**) and the Screw Vent Implant (TSV) (**B**), demonstrating the degree of bone apposition surrounding each implant.

**Figure 7:**
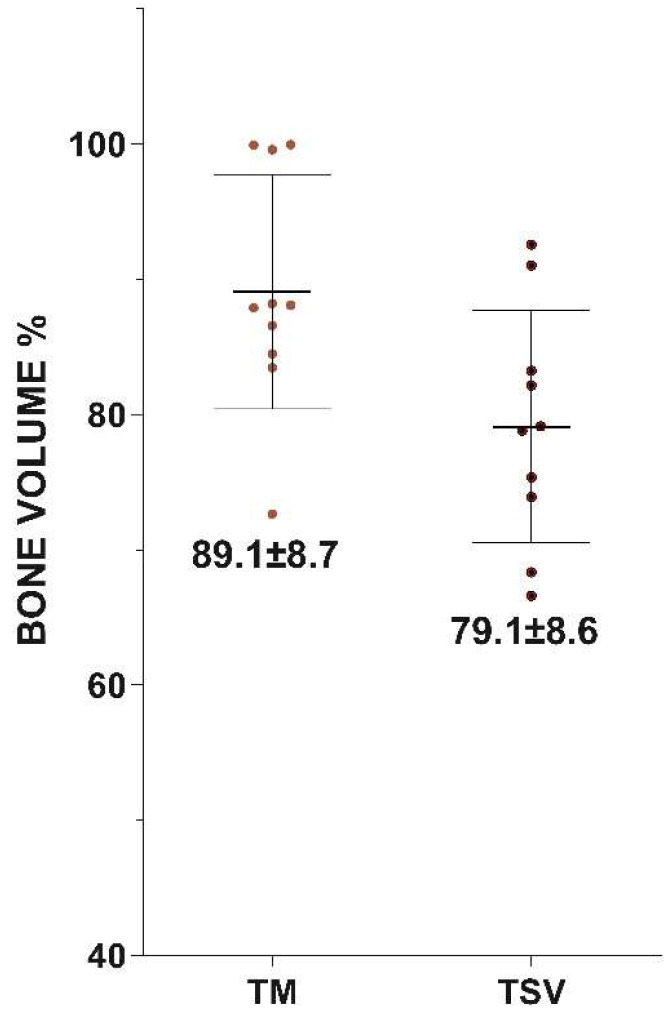
This figure presents a graph indicating the Bone Volume (BV) percentage at the region of interest surrounding both the Trabecular Metal Implant and the Screw Vent Implant (TSV), as determined by micro-CT.

Micro-CT Assessment: The findings from the bone volume (BV) evaluations conducted using micro-CT are illustrated in Figure 6. The average bone volume for TM implants was recorded as 89.1 ± 8.7, compared to 79.1 ± 8.6 for TSV implants. The percentage of bone volume surrounding the Trabecular Metal implant was markedly higher than that surrounding the TSV implants, as depicted in Figure 7.

## 4. Discussion

Dental implant success relies on osseointegration, which is influenced by various factors, including implant material, design, and surface properties [27,28]. The mechanical strength can also be altered by modifying the composition of the implant material used. For instance, modified titanium Ti-6Al-4V has demonstrated high shear bending resistance [29]. One approach to enhance osseointegration is using porous tantalum material, which has demonstrated success in implants [11]. The incorporation of porous tantalum in dental implants, such as a modified tapered, multithreaded titanium implant design, has shown promising outcomes [7,9,11]. A tapered, multithreaded, root-form, titanium dental implant design was modified by incorporating threads in the implant body’s midsection with an unthreaded sleeve of highly porous tantalum material [30].

Primary implant stability is pivotal in achieving successful osseointegration [31,32]. This stability minimizes implant micromotion, allowing for unhindered healing and osseointegration. The structure of porous tantalum metal offers high volumetric porosity, a low modulus of elasticity, and relatively high frictional characteristics [11,33]. The porous tantalum material’s high frictional properties may potentially enhance implant stability against the adjacent bone. This material has been used for over a decade to stabilize orthopedic implants through ingrowth [11,33,34]. Tantalum implants have demonstrated excellent biocompatibility and physical and mechanical properties well-suited for enhanced biological incorporation and structural integrity [14]. Tantalum has been widely used in the orthopedic and dental fields due to its corrosion resistance, fracture toughness, and biocompatibility [14,35]. Additionally, tantalum-based coatings have shown good antibacterial activity, essential for preventing peri-implant-related infections [36].

The tantalum in the midsection enhanced bone engagement, contributing to early implant stabilization [37] and subsequent ingrowth into the tantalum trabeculae. Other researchers have histologically verified the new bone formation around the tantalum mesh [5,38]. Histological observation of the tantalum mesh in the region of interest showed enhanced bone growth and osteocyte infiltration into the tantalum mesh region, suggesting that the Trabecular Metal material promotes bone ingrowth for secondary implant stability [38]. We found a larger quantity of peri-implant bone formation in close apposition to the tantalum region than to the titanium surfaces. The development of vascular canals and partial remodeling of these bone trabeculae were also observed. The bone trabeculae were densely populated with osteocyte lacunae, the majority of which were globular and irregularly spaced, characteristics of woven bone.

The bone-to-implant contact and bone volume percentages in this study were higher with the Trabecular Metal implants, aligning with observations made by Kim et al. [5]. The increased bone-to-implant contact and bone volume could be attributed to the rough implant surface of the TM implants. Several in vitro studies [39], histologic studies in animals [40,41] and humans, and clinical investigations [42,43] have demonstrated the superiority of rough surfaces over smooth surfaces regarding early bone-to-implant contact percentages. Studies have established a positive correlation between the roughness of the implant surface and the success of implant integration in bone tissue [20,27,44]. Increased surface roughness enhances cell adhesion, proliferation, and differentiation [45]. Another advantage of roughened titanium surfaces is a shorter healing period with a good prognosis due to better bone anchorage [24,46]. An implant system based on additively manufactured Ti-42Nb has emerged as a potential alternative for implant materials. Its high mechanical strength, coupled with a low elastic modulus, makes Ti-42Nb an intriguing material choice for orthopedic and dental implants [47]. Cooper [48] concluded that increasing the surface roughness of titanium implants improved bone integration with more bone formation at the interface by osteoconduction and osteogenesis.

New Zealand Rabbits were selected for this study based on several factors. The sample size was ascertained from the data from previous studies that were conducted at our center and following the 3 Rs principle [23]. These animal models offer the opportunity for observing implant stability and histological observations, allowing for measuring bone-to-implant contact and bone volume [24,49]. Controlled quantitative histological studies in humans are not feasible. The animal model in the present experiment offered the opportunity to place two different types of dental implants in the femur area using the same surgical procedure. Rabbits show a faster skeletal change and bone turnover rate than large animal models such as goats, dogs, and humans. The bone formation around implants in rabbits is much quicker than in humans. The length of the bone remodeling cycle is six weeks in rabbits compared with about four months in humans [50,51]. The observed differences in bone remodeling rates between rabbits and humans underscore the importance of cautious interpretation and application of animal study results to human health scenarios. This suggests that while rabbits serve as valuable models for initial testing and understanding of bone formation around implants, the speedier process in rabbits may not accurately replicate the complexities and timeline of human bone remodeling [52].

The quicker bone remodeling cycle in rabbits may offer researchers a faster initial insight into the efficacy and safety of new implant materials or techniques. However, this rapid remodeling could also mask potential problems that might surface over a more extended period, as seen in humans. Therefore, findings from rabbit studies should be complemented by subsequent research in animals with slower remodeling rates or directly in humans when ethically permissible and safe. In future research, this discrepancy in remodeling rates could be taken into account while designing studies, perhaps by monitoring rabbit bone remodeling over a longer term or by employing a series of models that more closely mimic human bone remodeling timelines [53]. Long-term follow-up studies in humans would also be crucial for validating findings from animal models. This could lead to a more comprehensive understanding of bone–implant interactions and ultimately improve the outcomes and lifespan of implant procedures in humans [54].

Some limitations of this study include that mechanical loading of the implant was not performed, even though it induces substantial changes in histomorphology at the bone–implant interface and mechanical stability of the whole implant system. Additionally, the human oral environment might realistically represent actual healing conditions.

## 5. Conclusions

The findings from our research underscore the potential effectiveness of Trabecular Metal (TM) dental implants in bolstering osseointegration and implant stability. Comparative analyses of histological and micro-CT data between TM and Traditional Screw Vent (TSV) implants demonstrated significantly improved bone-to-implant contact (BIC) and bone volume (BV) percentages for TM implants. This suggests that incorporating tantalum mesh in the TM implant design has led to enhanced osseointegration and bone ingrowth, resulting in the superior overall performance of the implant. This evidence implies that the TM implant design may be an instrumental breakthrough in dental implantology, offering improved stability and integration with host bone tissue. Nonetheless, it is crucial to emphasize that while these preliminary results are promising, additional investigations are required to confirm these outcomes across a larger sample size and diverse animal models. Further evaluations to determine the long-term durability of these implants under functional loading conditions are also necessary.

## Figures and Tables

**Table 1:** Randomization and implant installation scheme used in the study. TM represents Trabecular Metal implants and TSV represents Traditional Screw Vent implants.

Rabbit Serial No	Right Femur	Left Femur
SA-01	TM	TSV
SA-02	TSV	TM
SA-03	TM	TSV
SA-04	TSV	TM
SA-05	TM	TSV
SA-06	TSV	TM
SA-07	TM	TSV
SA-08	TSV	TM
SA-09	TM	TSV
SA-10	TSV	TM

## Data Availability

The data presented in this study are available on request from the corresponding author. The data are not publicly available due to the department policy.
